# IsoSVM – Distinguishing isoforms and paralogs on the protein level

**DOI:** 10.1186/1471-2105-7-110

**Published:** 2006-03-06

**Authors:** Michael Spitzer, Stefan Lorkowski, Paul Cullen, Alexander Sczyrba, Georg Fuellen

**Affiliations:** 1 Division of Bioinformatics, Biology Department, Schlossplatz 4, 48149 Münster, Germany; 2 Leibniz Institute of Arteriosclerosis Research, Domagkstr. 3, 48149 Münster, Germany; 3 Institute of Biochemistry, Wilhelm-Klemm-Str. 2, 48149 Münster, Germany; 4 Faculty of Technology, Research Group in Practical Computer Science, University of Bielefeld,Postfach 10 01 31, 33501 Bielefeld, Germany; 5 Department of Medicine, AG Bioinformatics, Domagkstr. 3, 48149 Münster, Germany

## Abstract

**Background:**

Recent progress in cDNA and EST sequencing is yielding a deluge of sequence data. Like database search results and proteome databases, this data gives rise to inferred protein sequences without ready access to the underlying genomic data. Analysis of this information (e.g. for EST clustering or phylogenetic reconstruction from proteome data) is hampered because it is not known if two protein sequences are isoforms (splice variants) or not (i.e. paralogs/orthologs). However, even without knowing the intron/exon structure, visual analysis of the pattern of similarity across the alignment of the two protein sequences is usually helpful since paralogs and orthologs feature substitutions with respect to each other, as opposed to isoforms, which do not.

**Results:**

The IsoSVM tool introduces an automated approach to identifying isoforms on the protein level using a support vector machine (SVM) classifier. Based on three specific features used as input of the SVM classifier, it is possible to automatically identify isoforms with little effort and with an accuracy of more than 97%. We show that the SVM is superior to a radial basis function network and to a linear classifier. As an example application we use IsoSVM to estimate that a set of *Xenopus laevis *EST clusters consists of approximately 81% cases where sequences are each other's paralogs and 19% cases where sequences are each other's isoforms. The number of isoforms and paralogs in this allotetraploid species is of interest in the study of evolution.

**Conclusion:**

We developed an SVM classifier that can be used to distinguish isoforms from paralogs with high accuracy and without access to the genomic data. It can be used to analyze, for example, EST data and database search results. Our software is freely available on the Web, under the name IsoSVM.

## Background

Typical eukaryotic genes are composed of several relatively short exons that are interrupted by long introns. The primary transcripts of most eukaryotic genes are composed of introns and exons separated by canonical splice sites. These mRNA precursors are shortened by a process called RNA splicing in which the intron sequences are removed yielding the mature transcript consisting of exons only [1]. However, cells can splice the primary transcript in different ways and thereby generate different polypeptides from the same gene (reviewed in [2]). This process is called alternative splicing. The different polypeptides are termed alternatively spliced gene products, splice variants or protein isoforms [3].

To generate correctly spliced, mature mRNAs, the exons must be identified and joined together precisely and efficiently by a complex process that requires the coordinated action of five small nuclear RNAs (termed U1, U2 and U4 to U6) and more than 60 polypeptides [3]. According to [3], five common modes of alternative splicing are known: (i) exon skipping or inclusion, (ii) alternative 3' splice sites, (iii) alternative 5' splice sites, (iv) mutually exclusive exons, and (v) intron retention which corresponds to no splicing. In complex pre-mRNAs, more than one of these modes of alternative splicing can apply to different regions of the transcript, and extra mRNA isoforms can be generated through the use of alternative promoters or polyadenylation sites [3].

Alternative splicing is a frequent process in eukaryotes. It is estimated that up to 60 percent of human genes are subjected to alternative splicing [3]. Thus, alternative splicing is probably an important source of protein diversity in higher eukaryotes. For example, the fruitfly *Drosophila melanogaster *contains fewer genes than *Caenorhabditis elegans *while exhibiting significantly higher protein diversity [2]. Furthermore, alternative splicing of primary transcripts is often tissue- or stage-specific (cf. the expression of different alternatively spliced transcripts during different stages of the development of an organism [4]), and is thus an important regulatory mechanism.

**Figure 1:**
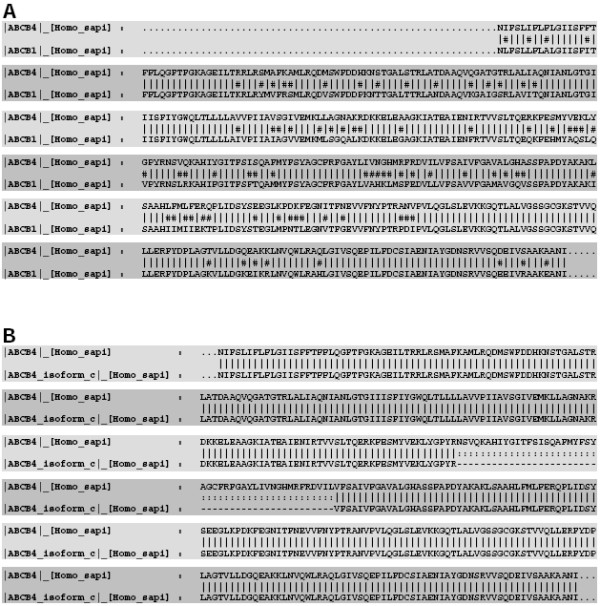
**Visualization of a part of an alignment of (A) **two paralogous sequences (the human ABCB4 and ABCB1 protein) and **(B) **two isoforms (the human ABCB4 protein and its isoform *c*), representing an ideal case. Positions with matches between the two sequences are indicated by "|", mismatches by "#" and amino acids vs. gap characters by ":". The values of the three features (cf. ***Methods***, section *Features*) for the *full-length *sequences compared in panel (A) are (i) *sequence similarity *75.76%, (ii) *inverse CBIN count *0.0027, (iii) *fraction of consecutive matches and mismatches *0.7111. For the *full-length *sequences compared in panel (B) we have (i) sequence similarity 96.33%, (ii) inverse CBIN count 0.3333, (iii) fraction of consecutive matches and mismatches 0.9969.

For a protein in an organism, other proteins can be found that are homologous, i.e. that are similar due to common evolutionary ancestry. Following Fitch [5], there can be orthologs, which are homologs due to a speciation event, and paralogs, which are homologs due to a duplication event. Even if genomic information on intron/exon-structure is not available, isoforms can usually be visually distinguished from homologs based on protein sequence alone, since only the latter feature substitutions with respect to each other (cf. Figure 1). For the remainder of this paper, without loss of generality, we will consider paralogs only. Comparing a protein with an isoform of its paralog, we still find a predominance of substitutions, and we consider these two proteins to be paralogs.

Available databases of proteins and their isoforms consider only a small number of protein families and species (see e.g. [6-8]). We wanted to identify isoforms without knowledge of genomic information and independently of specific protein families or species, in a fashion well suited for high-throughput genomics and proteomics.

**Figure 2:**
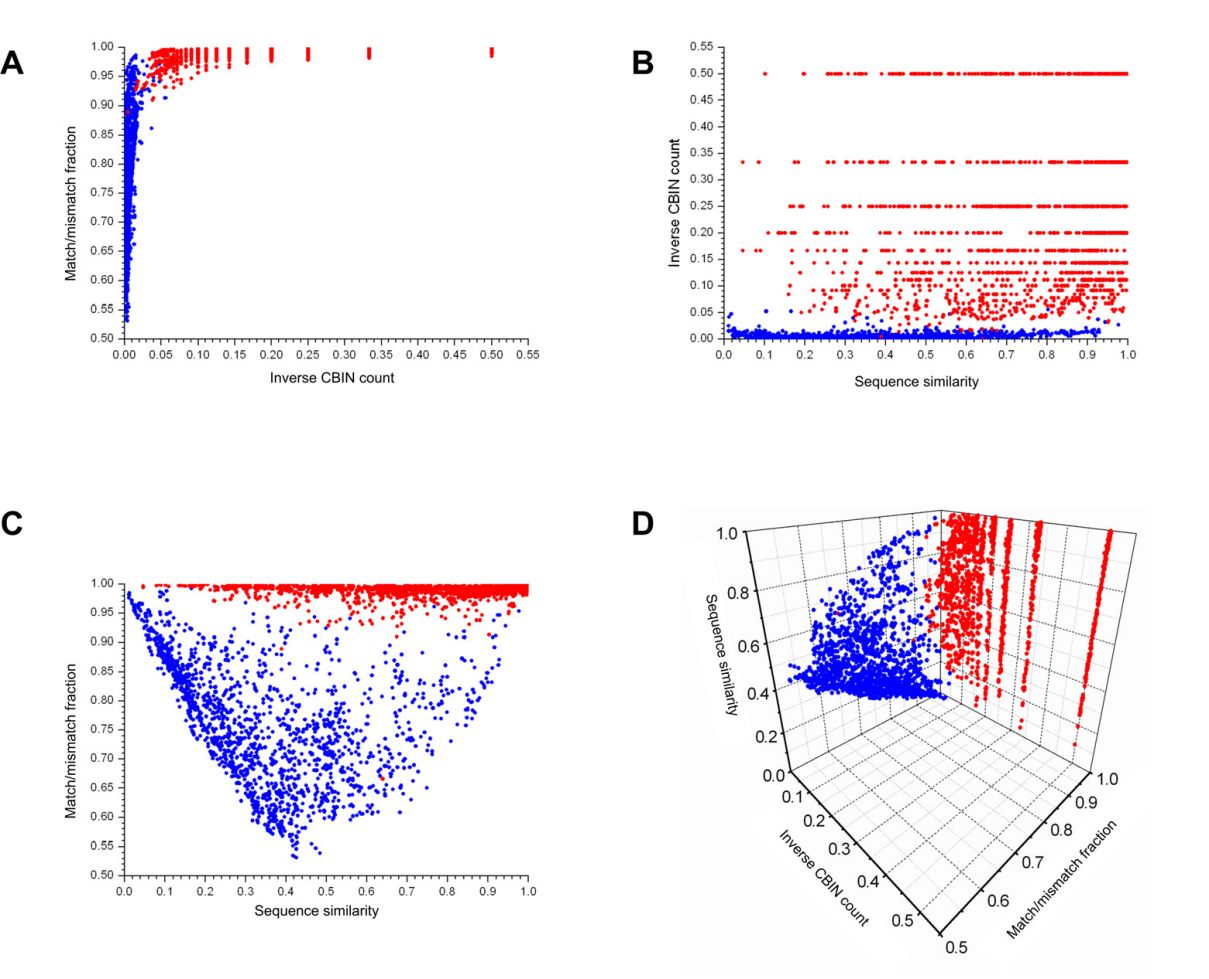
**Features displayed by the samples in the canonical training dataset. **Panels **(A) **to **(C) **illustrate combinations of two of the three features. Panel **(D) **illustrates all three features at the same time. Samples arising from the comparison of paralogous sequences are shown in blue, whereas isoforms are shown in red. An *inverse CBIN count *of *1*/*n *arises if *n *CBINs are featured by a given sample. Though the samples of both classes separate well in general, some samples of one class "overlap" into the other class.

**Figure 3:**
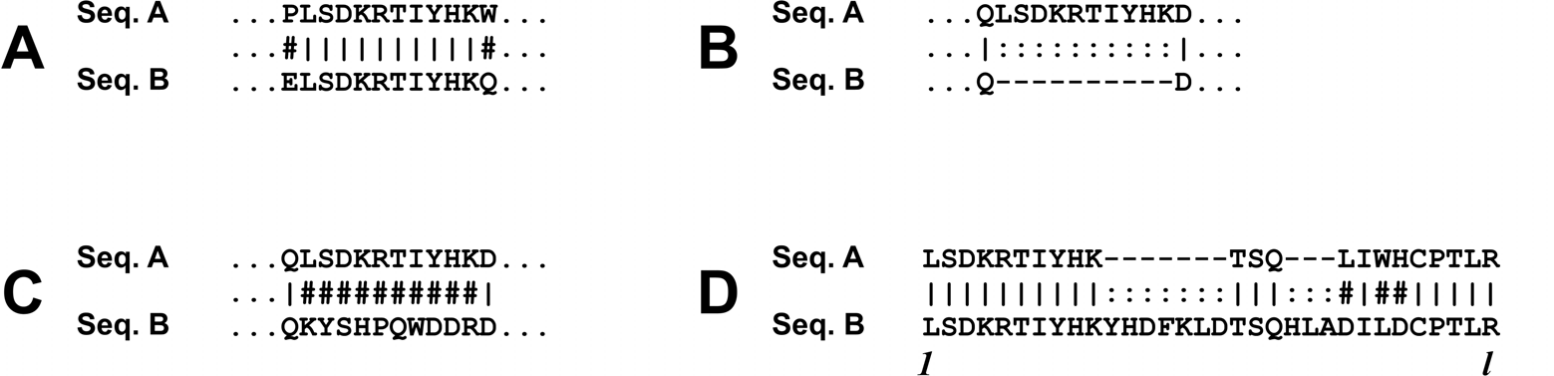
**Illustration of the different cases of consecutive blocks of identities or non-identities (CBINs). (A) **CBIN of matches, **(B) **CBIN of gaps (counted as mismatches), **(C) **CBIN of mismatches, **(D) **example of a comparison of two sequences with an alignment length of 32. Matches are denoted by "|", mismatches by "#" and amino acids aligned to gaps by ":". The example alignment of length 32 features eight CBINs. The values of the three features are: (i) sequence similarity 0.594, (ii) inverse CBIN count 0.125, (iii) fraction of consecutive matches and mismatches 0.75.

Visual inspection of large datasets such as complete proteomes (meaning the totality of all proteins expressed in an organism) would be time-consuming and prone to misclassifications. To enable automation, a set of three different features was derived based on the pairwise alignment of the two protein sequences to be compared. These features take into account such parameters as the distribution of substitutions and sequence similarity. The three features are *overall sequence similarity*, the *number of consecutive blocks of identities or non-identities *(CBINs) and the *overall number of consecutive matches (and mismatches)*, see also Figures 2 and 3, and ***Methods***, section *Features*.

For automation the approach of supervised learning using a Support Vector Machine (SVM) [9-11] was chosen. SVMs are gaining popularity in Bioinformatics [12-15] and are often superior to Neural Networks and Bayesian Learning [16]. SVM classifiers distinguish two classes of input data by calculating separating hyperplanes (decision surfaces) in a vector space *V *that is endowed with a dot product. The dot product is used as a measure of similarity. Data samples from the *input space *are mapped to the vector space *V *(usually of dimensionality higher than the input space), making it easier to find a separating hyperplane. The position and margin of the hyperplane are optimized in *V*, maximizing the distance of the hyperplane to instances of both classes. The kernel function used to measure similarity behaves in input space like the dot product in space *V*. Thus, similarity of input data can be measured easily in *V*. Without a kernel function, computation of the dot products in *V *would be necessary, consuming a large amount of time, depending on the structure of *V*. For an in-depth description of properties and theory of SVMs, please see [11]. The Support Vector Machine implementation SVMLight [17] was used. In this paper, we introduce a highly accurate SVM-based method to distinguish between isoforms and paralogs on the protein level (that is, without the need for genomic information). Our software is freely available on the Web (see **Conclusions**).

## Results and discussion

### Importance of maximizing accuracy in distinguishing isoforms and paralogs

Why does isoform detection require such a high degree of accuracy? Why do we want to use an SVM even though this approach is usually employed in case the input space has dimensionality (much) larger than three? For example, when performing 2,000 sequence comparisons, even a 0.2% improvement in accuracy results in 4 fewer misclassifications. Such numbers are typical, for example, in applications of our automated phylogeny pipeline RiPE [18,19]. Analyzing a large protein family with RiPE, few misclassifications make a difference since paralogs misidentified as isoforms (false positives) are deleted from the dataset, which may result in the loss of key members of the protein family, compromising the interpretation of the evolution of sequence, domain structure and function. (In this specific application, isoforms misidentified as paralogs (false negatives) do not pose a major problem.)

### Performance statistics of different classifiers based on three features

**Figure 4:**
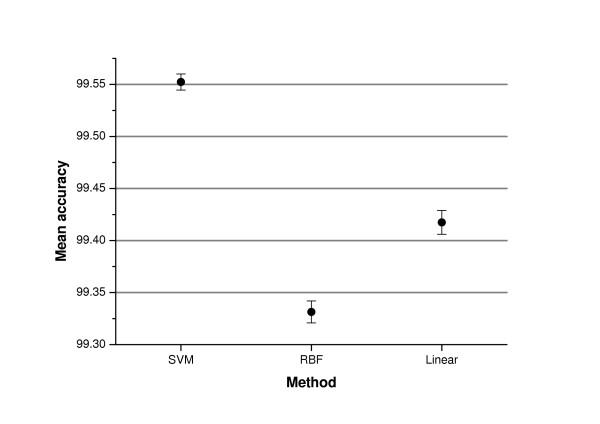
**Accuracy of classifiers measured by jackknife resampling, employing all three features. **Performance of the SVM classifier is compared to classifiers based on an RBF network as well as a linear classifier. Mean accuracy and standard error of the mean were assessed by 100-fold jackknife resampling using 7604 samples resulting from a visual inspection process of protein sequences taken from Genbank.

We investigated three different classifiers designed to distinguish isoforms and paralogs. We calculated the *mean accuracy *and *standard error of the mean *for an SVM, a radial basis function (RBF) network [20] and a linear classifier. Classification was based on three features and samples were derived from protein data taken from Genbank [21] (cf. ***Methods***, section *Assessing performance of classifiers based on three features by jackknife resampling*). The SVM classifier showed better accuracy and a smaller standard error of the mean than the two other classifiers. In detail, the SVM classifier shows a mean accuracy of 99.55% and a standard error of 0.008. In contrast, the classifier based on the RBF network shows a mean accuracy of 99.33% and a standard error of 0.011, while for the linear classifier a mean accuracy of 99.42% and a standard error of 0.011 was observed. Mean accuracy, mean precision and true positive/true negative (TP/TN) and false positive/false negative (FP/FN) numbers for the three classifiers are given in Table 1 and illustrated in Figure 4.

### Performance of different classifiers using a canonical training/testing dataset

In the following, we report results that are not supported by resampling but derived from a specific ("canonical") training and testing dataset (cf. ***Methods***, section *Canonical training and testing dataset*). In this way, we were able to explore, on a large (3802 samples) dataset, a wide variety of classifiers in reasonable time.

The SVM classifier distinguishes isoforms and paralogs of the canonical testing dataset with an accuracy of 99.63% and a precision of 99.37% (cf. Table 2 and 3). All three sequence-based features used by the SVM (cf. Figure 2) contributed to accuracy; results based on any combination of two features only were inferior, as shown in Table 3.

A linear classifier that was calculated using all three features of the samples in the canonical training dataset was found to classify the canonical testing dataset with an accuracy of 99.42%. Linear classifiers that were trained using all possible combinations of only two features showed at least slightly inferior results compared to the linear classifier based on all three features. Not surprisingly, the best-performing classifier based on two features does not use the weakest feature that is *sequence similarity*. Classifiers based on *sequence similarity *alone appear to be weak in distinguishing between isoforms and paralogs and perform much worse than any other of the tested classifiers; a linear classifier derived by line-sweeping using the feature *sequence similarity *alone results in an accuracy of approximately 82%. Linear classifiers based on one of the other features perform surprisingly well, however (cf. Table 3).

Finally, the radial basis function (RBF) network classifier [20] (cf. ***Methods*, **section *Training of the radial basis function network*) applied to the canonical testing dataset using all three features results in an accuracy of 99.32%.

### Application of the SVM classifier to EST data

As a first real-life application we used IsoSVM to search for isoforms within the CAP3-derived contigs of 722 *Xenopus laevis *EST clusters [22]. *Xenopus laevis*, as an allotetraploid species, has undergone a genome wide duplication. Therefore, many genes are represented by two paralogs. Isoforms of *X*. *laevis *proteins have not been studied yet in any systematic way. Sequencing the *X. laevis *genome is made difficult by its sheer size, and genomic sequence data are too few in number to study intron-exon structures of most genes. Contigs were derived from 350,468 *Xenopus *ESTs downloaded from GenBank. After cleanup of the EST data (high quality sequence clipping, vector and repeat masking), sequences were clustered using an enhanced suffix array based approach [23] implemented in the tool Vmatch [24]. Clustering resulted in 25,971 clusters which were assembled into 31,353 contigs using CAP3 [25]. Table 4 summarizes the results of the clustering process.

To assess whether the splitting of clusters by CAP3 into several contigs was caused by grouping isoforms into the same cluster, or whether the splitting was due to paralogs, we extracted 722 clusters that have multiple contigs (2,243 contigs total), and for which each contig has a full length protein match in the protein NR database [21]. Most of the 722 clusters consist of only two contigs and only a fraction features three or more contigs. Treating each contig consensus as a sequence, 5,459 sequence pairs were compared by IsoSVM within clusters; 986 of these samples (19.3%) were classified as isoforms and 4,125 as paralogs (80.7%). 348 samples were left out, representing contigs with almost no overlap, i.e. sequence pairs of low (<1%) similarity. As a further check, to assess the accuracy of this analysis, 290 randomly chosen samples were reviewed manually and the result was noted (cf. Table 2); an accuracy of 97.93% and a precision of 99.23% was found. (In a few cases, early EST sequencing termination events produce a block of amino acids aligned with gaps at the end of the two sequences compared, causing classification of such cases as isoforms, and they were counted as such.)

### Application of the SVM classifier to an automated phylogeny pipeline

As a second application, the classifier was incorporated into a pipeline for automatic generation of protein phylogenies called RiPE [18,19], with the aim to further reduce the redundancy of the RiPE-retrieved protein data by recognizing and deleting sequences that are isoforms. Isoforms are usually considered irrelevant data in phylogenetic tree inference and analysis. RiPE data are generated by homology search (PSIBLAST, [26]), retrieving hits with putative homology to a search profile and assembling HSP-based homologous-regions-only data as described in ***Methods*, **section *Homologous regions only*. The pipeline already features a redundancy minimization stage, sorting out hits that are similar to other hits (95% identity or more). The IsoSVM classifier was incorporated, enabling the detection and deletion of isoforms, thus decreasing dataset size and redundancy while simultaneously increasing computational speed and legibility of the phylogenetic tree. We first tested the ability of our classifier to deal with homologous-regions-only data (using the testing dataset described in ***Methods***, section *Homologous regions only*), noting an accuracy of 98.98% and a precision of 97.57% (cf. Table 2). Training on homologous-regions-only data did not improve classifier performance (data not shown).

Following our interest in ABC (**A**TP-**b**inding **c**assette) proteins, which are found in a wide variety of species and are of major biomedical importance, a dataset of 1,349 ABC protein hits was then retrieved by RiPE from 20 model proteomes (12 eukaryotes, 6 bacteria and 2 archaea) using 48 known human ABC proteins [27] as search profile. 115 hits were identified as isoforms of another hit by the SVM classifier. As a further check, all 115 putative isoforms were inspected visually, the automatic classification (isoform or paralog) was checked, and a precision of 95.65% was found. The accuracy of the classifier was not calculated in this case since RiPE reports only samples classified as positives (i.e. isoforms). While the precision reported is based on the number of false positives (i.e. sequences representing paralogous sequences being reported as isoforms), assessment of accuracy would require the visual inspection of tens of thousands of samples of (putative) paralogs, i.e. putative false negatives. Removal of isoforms resulted in a reduction of dataset size by about 8%, rendering the eukaryotic parts of the tree much more legible.

### Limitations of the classifier

Despite showing reliable performance, the SVM classifier is not perfect. It may misleadingly classify a small portion of paralogs with high similarity as isoforms, since they feature long stretches of identical amino acid sequence. Further, sequences that are fragments of other sequences will be classified as isoforms.

## Conclusion

The SVM classifier, trained using visually classified cases of isoform and paralog relationships, proved to be reliable in all tests, exhibiting an accuracy of over 97% and a precision of over 95%. We are thus able to distinguish isoforms and paralogs in a satisfactory way, no matter whether full-length, homologous-regions-only or EST cluster sequences are handled. In particular, for species such as *Xenopus laevis*, for which few detailed analyses of the evolution of genes and proteins exist, the analysis of paralogs and isoforms can improve statistical models of sequence evolution, e.g. regarding the likelihood of gene duplication and alternative splicing. Overall, the IsoSVM tool should be useful for researchers in several fields of genomic research and EST analysis as a reliable method of automatic isoform identification. Our software is freely available at the IsoSVM Website [28], under an open source license.

## Methods

To automatically determine if one protein sequence is an isoform of another, we first derive three features, characterizing the degree and pattern of matches and mismatches in a pairwise alignment of the two sequences as detailed in the paragraphs below. The three features depend on the length of the alignment of the two sequences and **c**onsecutive **b**locks of **i**dentities or **n**on-identities (CBINs).

### Prerequisites

#### Length of the alignment (*l*)

The length of the alignment of two protein sequences *a *and *b *is used in two of the features described below to normalize their values to a range from 0 to 1. This was done in order to avoid numerical problems that may affect classification performance and to exclude features of large absolute amount that may numerically dominate smaller ones during training of the SVM (cf. [29,30]).

#### Consecutive blocks of identities or non-identities (CBIN)

A CBIN is a block in which the alignment features consecutive matches or mismatches (cf. Figure 3). Few large CBINs are characteristic for comparisons of isoforms whereas many short CBINs are typically found in comparisons of paralogs (cf. Figure 1, illustrating the comparison of two isoforms and two paralogs).

There are two possible cases of a CBIN. First, if sequence *a *features a subsequence of length *c *starting at position *i *(with *c *between 1 and *l-i*) that is a maximum run of exact matches (that cannot be extended any further) to its aligned counterpart of sequence *b*, then this block of consecutive matches is a CBIN of length *c*. Second, if sequence *a *features a subsequence of length *c *starting at position *i *(with *c *between 1 and *l-i*) that is a maximum run of mismatches to its aligned counterpart of sequence *b*, then this block of consecutive mismatches is a CBIN of length *c*. Formally, for internal CBINs that are not located at the beginning or at the end of the alignment, we have

*a*_*k *_= *b*_*k *_for all *k*,*k *= *i*,...,*i*+*c *    **and **    *a*_*i*-1 _≠ *b*_*i*-1 _    **and **    *a*_*i*+*c*+1≠ _*b*_*i*+*c*+1 _

or

*a*_*k*_≠ *b*_*k*_for all *k*,*k *= *i*,...,*i*+*c *    **and **    *a*_*i*-1 _= *b*_*i*-1 _    **and **    *a*_*i*+*c*+1 _= *b*_*i*+*c*+1 _    (1)

where *i *is the start coordinate and *i+c *the end coordinate of the maximum block of matches or mismatches. For CBINs that are not internal, the definition can be generalized in an obvious way. Amino acids aligned with gaps are considered mismatches.

### Features

#### Sequence similarity

Sequence similarity is the overall number of matches in the alignment of the sequences *a *and *b*, divided by its length *l*:

Feature 1=|{i,i=1,…,l|ai=bi}|l,     (2)
 MathType@MTEF@5@5@+=feaafiart1ev1aaatCvAUfKttLearuWrP9MDH5MBPbIqV92AaeXatLxBI9gBaebbnrfifHhDYfgasaacH8akY=wiFfYdH8Gipec8Eeeu0xXdbba9frFj0=OqFfea0dXdd9vqai=hGuQ8kuc9pgc9s8qqaq=dirpe0xb9q8qiLsFr0=vr0=vr0dc8meaabaqaciaacaGaaeqabaqabeGadaaakeaacqqGgbGrcqqGLbqzcqqGHbqycqqG0baDcqqG1bqDcqqGYbGCcqqGLbqzcqqGGaaicqaIXaqmcqGH9aqpdaWcaaqaamaaemaabaGaei4EaSNaemyAaKMaeiilaWIaemyAaKMaeyypa0JaeGymaeJaeiilaWIaeSOjGSKaeiilaWIaemiBaWMaeiiFaWNaemyyae2aaSbaaSqaaiabdMgaPbqabaGccqGH9aqpcqWGIbGydaWgaaWcbaGaemyAaKgabeaakiabc2ha9bGaay5bSlaawIa7aaqaaiabdYgaSbaacqGGSaalcaWLjaGaaCzcamaabmaabaGaeGOmaidacaGLOaGaayzkaaaaaa@56F3@

where |*M*| denotes the number of elements in a set *M*.

#### Inverse CBIN count

As the second feature we us the reciprocal value of the number of CBINs *n *in the pair of aligned sequences:

Feature 2=1n.     (3)
 MathType@MTEF@5@5@+=feaafiart1ev1aaatCvAUfKttLearuWrP9MDH5MBPbIqV92AaeXatLxBI9gBaebbnrfifHhDYfgasaacH8akY=wiFfYdH8Gipec8Eeeu0xXdbba9frFj0=OqFfea0dXdd9vqai=hGuQ8kuc9pgc9s8qqaq=dirpe0xb9q8qiLsFr0=vr0=vr0dc8meaabaqaciaacaGaaeqabaqabeGadaaakeaacqqGgbGrcqqGLbqzcqqGHbqycqqG0baDcqqG1bqDcqqGYbGCcqqGLbqzcqqGGaaicqqGYaGmcqGH9aqpdaWcaaqaaiabigdaXaqaaiabd6gaUbaacqGGUaGlcaWLjaGaaCzcamaabmaabaGaeG4mamdacaGLOaGaayzkaaaaaa@3FB7@

#### Fraction of consecutive matches and mismatches

This feature describes the overall number of consecutive matches and mismatches (not counting the match or mismatch at the first position of a CBIN). In other words, it is the sum of the lengths *c*_*j *_minus one, of all *n *CBINs (with *j = 1..n*), divided by *l*:

Feature 3=∑j=1n(cj−1)l.     (4)
 MathType@MTEF@5@5@+=feaafiart1ev1aaatCvAUfKttLearuWrP9MDH5MBPbIqV92AaeXatLxBI9gBaebbnrfifHhDYfgasaacH8akY=wiFfYdH8Gipec8Eeeu0xXdbba9frFj0=OqFfea0dXdd9vqai=hGuQ8kuc9pgc9s8qqaq=dirpe0xb9q8qiLsFr0=vr0=vr0dc8meaabaqaciaacaGaaeqabaqabeGadaaakeaacqqGgbGrcqqGLbqzcqqGHbqycqqG0baDcqqG1bqDcqqGYbGCcqqGLbqzcqqGGaaicqaIZaWmcqGH9aqpdaWcaaqaamaaqahabaGaeiikaGIaem4yam2aaSbaaSqaaiabdQgaQbqabaGccqGHsislcqaIXaqmcqGGPaqkaSqaaiabdQgaQjabg2da9iabigdaXaqaaiabd6gaUbqdcqGHris5aaGcbaGaemiBaWgaaiabc6caUiaaxMaacaWLjaWaaeWaaeaacqaI0aanaiaawIcacaGLPaaaaaa@4C43@

The feature *fraction of consecutive matches and mismatches *is abbreviated as *match-mismatch fraction *in all figures and tables. In the following we describe the procedure of the generation of the training and testing datasets, the learning pipeline and the validation of classifier performance.

### Generation of the training and testing datasets

#### Sequence retrieval, homology search and visual classification

The NCBI non-redundant (NR) database [21] was used as the source for retrieving protein sequences and was downloaded from the NCBI FTP server on March 8, 2004. The NR database was then searched for sequences annotated as "isoform" or "splice variant". 13,061 sequences featuring at least one of the two keywords were found and retrieved from the NR database, establishing a set of unrelated sequences that are from any species for which isoforms can be expected to exist. From this set, 250 sequences were randomly selected to give rise to the canonical training and testing datasets, as follows (for a complete list of taxa included in this set please consult the supplementary material [see Additional file 1]).

**Figure 5:**
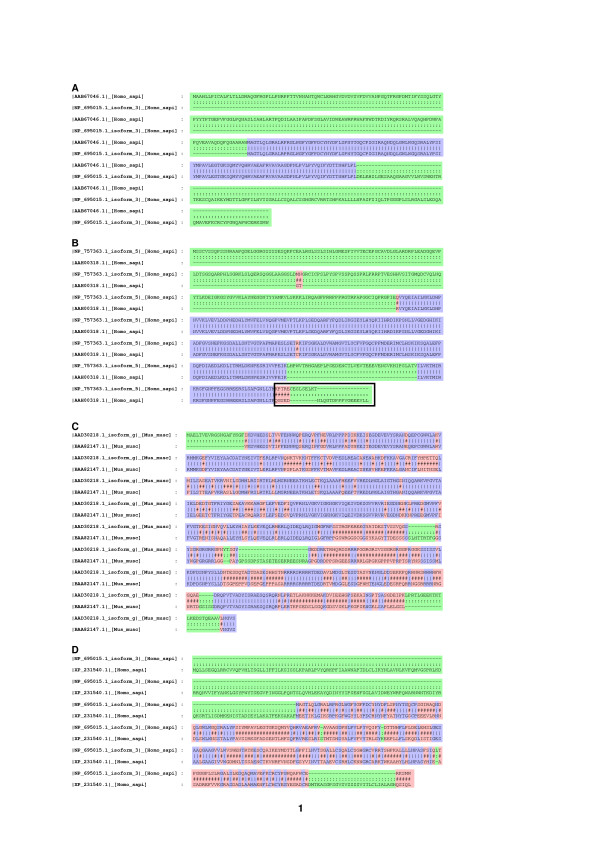
**Visual inspection process. **Matches in the alignments are colored in blue and mismatches in red. Amino acids aligned to gaps are indicated in green. Panels **(A) **to **(D) **illustrate alignments of two protein sequences classified as isoforms (panels **(A) **and **(B)**) or as paralogs (panels **(C) **and **(D)**). The sequences shown in panel **(A) **feature a shared subsequence (a putative constitutive exon), marked in blue. The upper sequence features an additional exon at the beginning (marked in green) that is missing in the lower sequence. In contrast, a putative exon at the end (also shown in green) is found in the lower sequence only. Comparison of the two putative isoforms shown in panel **(B) **reveals two constitutive exons in the middle and towards the end of the alignment, colored in blue (the only mismatch is interpreted as a sequencing error, or a polymorphism). These are separated by a stretch of amino acids aligned to gaps, interpreted as an exon skipped in the lower sequence. At the beginning of the alignment, the upper sequence features a long stretch of amino acids aligned to gaps and a few mismatches; two mutually exclusive exons are a plausible interpretation, since the lower sequence (starting with G and not with M) is incomplete and its first exon is probably much longer. At the end of the alignment both sequences feature a stretch of mismatches and gaps (colored in red), interpreted as mutually exclusive exons (indicated by a black frame). The sequences compared in panel **(C) **give rise to a sample of the paralog class. In general, the alignment features many mismatches, interpreted as substitutions, and six stretches of amino acids aligned to gaps (putative deletions). Panel **(D) **illustrates another putative paralog. Besides a shared stretch (featuring numerous substitutions) in the middle of the alignment, the upper sequence features putative deletions, or missing exons. It may thus be a case of an isoform of a paralog.

For all 250 sequences a BLAST search [26] was performed, again on the NR database, using each sequence as the query sequence. BLAST standard parameters and an E-value threshold of 10^-90^ were used to ensure that no unrelated hits were retrieved. For 176 of the 250 query sequences, hits corresponding to putative homologous sequences or isoforms were found. All sequences corresponding to hits from the same species as the query were retrieved from the NR database in full length. Sequences were then aligned using the program *fftnsi *of the MAFFT package [31] using default values (PAM200 log-odds matrix [32], gap open penalty 2.4, gap extension penalty 0.06). The resulting multiple alignment gives rise to pairwise alignments of all pairs of sequences. We obtained each pairwise alignment from a multiple alignment to improve the quality of the pairwise alignment (see e.g. [33]). Finally, each pair of sequences was assigned to one out of two possible classes (+1,-1) based on visual inspection (cf. Figure 5). A value of +1 indicates isoforms and a value of -1 paralogs. A few cases where no clear decision was possible and sequence pairs of low similarity (<1%) were not included in the data. More specifically, two sequences are classified as isoforms if their alignment displays the following evidence:

1. We observe large blocks of (almost) identical sequence with no (or few) mismatches that can be interpreted as common exons, except for a few sequencing errors or polymorphisms.

2. Additionally, we observe either one or both of the following:

i. We observe one or more sequence blocks that do not match (interspersed with a few random matches) which can be interpreted as mutually exclusive exons of similar size that are spuriously aligned and which are embedded in blocks of (almost) identical sequence.

ii. We observe one or more sequence blocks that align to gap characters which can be interpreted as surplus amino acids that arise if mutually exclusive exons of different length are spuriously aligned, or if exon(s) are missing in one of the sequences, or if an exon has an alternative splice site such that it is observed in a short and in a long version, and which are again embedded in blocks of (almost) identical sequence.

In contrast, two sequences are classified as paralogs if there is a large sequence block that displays sufficient similarity to allow assumption of common evolutionary origin, interspersed with a sufficiently large number of mismatches that must be interpreted as substitutions and that cannot be interpreted as sequencing errors, etc. Paralogs may feature deletions that give rise to observations similar to the ones in (i) and (ii) which are however embedded in blocks of sufficient similarity with many mismatches.

#### Canonical training and testing dataset

The dataset resulting from visual inspection featured 3,802 samples of the isoform class and 8,757 of the paralog class. We started training with many more paralogs than isoforms, with inferior testing results (data not shown). Therefore, to prevent one class from outweighing the other during SVM training, the number of samples of the larger class was truncated to 3,802 samples. One half of the dataset, consisting of 1,901 isoform and 1,901 paralog samples, was designated the canonical training dataset, the other half is the canonical testing dataset. As can be seen from Figure 2, the two classes separate quite well, although close inspection reveals that the boundary between them is in fact quite complex.

#### Homologous regions only

Another testing dataset was generated directly from the database search reports obtained before. They were converted into FASTA-formatted alignments of merged HSPs (partial hits called *high-scoring segment pairs*) using MVIEW [34]. These merged HSPs can be viewed as the concatenation of the homologous regions of the full hit sequences. Some of the queries contained internal repeats that do not give rise to a single concatenation; these sequences were left out. By automatically transferring the visual classification of the corresponding full-length-sequence-based samples above to the merged HSP data, a set of 8,066 classified samples was obtained (5,518 samples of the paralog and 2,548 samples of the isoform class).

### Training of the SVM

To find an optimum SVM classifier for a given problem, a kernel has to be specified. As kernel function the radial basis function (RBF) kernel was used. For SVMs with RBF kernels, two parameters, *C *and *g *need to be determined. *C *describes a penalty for training errors and is part of the soft margin concept of SVMs. It allows for a number of (misclassified) training samples to be located within the margin. Thus, a certain amount of noise is tolerated in the training data. The parameter *g *describes the width of the Gaussian bells of the radial basis function of the RBF kernel

K(xi,xj)=exp⁡(−g‖xi−xj‖2),g>0,     (5)
 MathType@MTEF@5@5@+=feaafiart1ev1aaatCvAUfKttLearuWrP9MDH5MBPbIqV92AaeXatLxBI9gBaebbnrfifHhDYfgasaacH8akY=wiFfYdH8Gipec8Eeeu0xXdbba9frFj0=OqFfea0dXdd9vqai=hGuQ8kuc9pgc9s8qqaq=dirpe0xb9q8qiLsFr0=vr0=vr0dc8meaabaqaciaacaGaaeqabaqabeGadaaakeaacqWGlbWscqGGOaakcqWG4baEdaWgaaWcbaGaemyAaKgabeaakiabcYcaSiabdIha4naaBaaaleaacqWGQbGAaeqaaOGaeiykaKIaeyypa0JagiyzauMaeiiEaGNaeiiCaa3aaeWaaeaacqGHsislcqWGNbWzdaqbdaqaaiabdIha4naaBaaaleaacqWGPbqAaeqaaOGaeyOeI0IaemiEaG3aaSbaaSqaaiabdQgaQbqabaaakiaawMa7caGLkWoadaahaaWcbeqaaiabikdaYaaaaOGaayjkaiaawMcaaiabcYcaSiabdEgaNjabg6da+iabicdaWiabcYcaSiaaxMaacaWLjaWaaeWaaeaacqaI1aqnaiaawIcacaGLPaaaaaa@539F@

where *x*_*i*_, *x*_*j *_denote feature vectors of training samples. We scanned for best parameter values in a specific range using a so-called grid-search.

**Figure 6:**
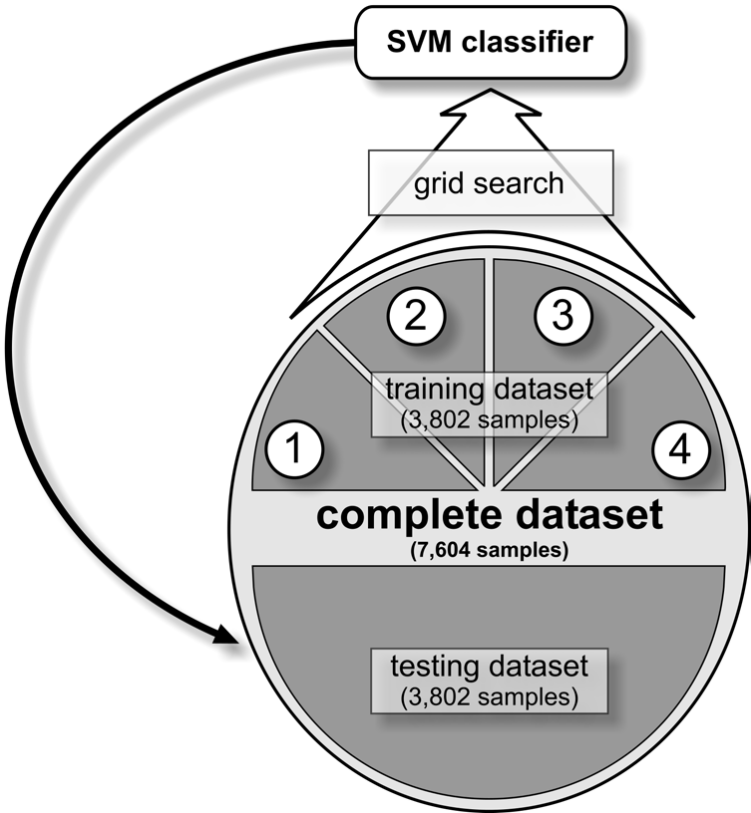
**SVM training process. **The complete dataset generated by visual inspection was split into two parts, yielding a canonical training dataset of 3,802 samples and a canonical testing dataset of 3,802 samples, each consisting of an equal number of isoform and paralog instances. The canonical training dataset was again split into four subsets (denoted by numbers in circles) and submitted to the grid-search procedure. The resulting classifier was then tested on the canonical testing dataset.

The grid-search was carried out for parameter *C *ranging from 10^-5 ^to 10^15 ^and for parameter *g *ranging from 10^-15^ to 10^3^, following [28]. Both parameters were scanned using 10 steps per axis on a logarithmic scale, resulting in a total number of 100 grid points. The grid-search was based on a cross-validation procedure intended to prevent overfitting of the classifier on the canonical training dataset, again following [28]. We split the training dataset into *n *= 4 subsets (cf. Figure 6, each subset is denoted by an encircled number). For each point of the grid evaluated by the grid-search, n-1 of the *n *subsets are used to train a classifier using the kernel parameters *C *and *g *corresponding to the point in the grid. The resulting classifier is then tested on the one remaining subset of the training dataset, and accuracy is recorded. The overall accuracy of the SVM classifier trained at a specific point of the grid is then the mean over all *n *accuracies. The maximum accuracy was identified and the corresponding kernel parameters *C *and *g *were noted. New parameter ranges (10^-1^-10^3 ^for C, 10^-2^-10^3 ^for *g*) were then used to run a second grid-search with higher resolution in the area in which maximum accuracy was found. Inside this new grid, the point of maximum mean accuracy (99.58%) was chosen and its corresponding kernel parameters (*C *= 12.5; *g *= 6.25) were noted. Final training was then carried out on the entire canonical training dataset, resulting in a final SVM classifier. To assess its performance true positive/true negative (TP/TN) and false positive/false negative (FP/FN) ratios were tallied and accuracy

TP+TN(TP+TN+FP+FN)     (6)
 MathType@MTEF@5@5@+=feaafiart1ev1aaatCvAUfKttLearuWrP9MDH5MBPbIqV92AaeXatLxBI9gBaebbnrfifHhDYfgasaacH8akY=wiFfYdH8Gipec8Eeeu0xXdbba9frFj0=OqFfea0dXdd9vqai=hGuQ8kuc9pgc9s8qqaq=dirpe0xb9q8qiLsFr0=vr0=vr0dc8meaabaqaciaacaGaaeqabaqabeGadaaakeaadaWcaaqaaiabdsfaujabdcfaqjabgUcaRiabdsfaujabd6eaobqaaiabcIcaOiabdsfaujabdcfaqjabgUcaRiabdsfaujabd6eaojabgUcaRiabdAeagjabdcfaqjabgUcaRiabdAeagjabd6eaojabcMcaPaaacaWLjaGaaCzcamaabmaabaGaeGOnaydacaGLOaGaayzkaaaaaa@4395@

and precision (cf. [35])

TP(TP+FP)     (7)
 MathType@MTEF@5@5@+=feaafiart1ev1aaatCvAUfKttLearuWrP9MDH5MBPbIqV92AaeXatLxBI9gBaebbnrfifHhDYfgasaacH8akY=wiFfYdH8Gipec8Eeeu0xXdbba9frFj0=OqFfea0dXdd9vqai=hGuQ8kuc9pgc9s8qqaq=dirpe0xb9q8qiLsFr0=vr0=vr0dc8meaabaqaciaacaGaaeqabaqabeGadaaakeaadaWcaaqaaiabdsfaujabdcfaqbqaaiabcIcaOiabdsfaujabdcfaqjabgUcaRiabdAeagjabdcfaqjabcMcaPaaacaWLjaGaaCzcamaabmaabaGaeG4naCdacaGLOaGaayzkaaaaaa@3A0B@

were calculated.

### Training of the radial basis function network

To compare the performance of the SVM classifier to another machine learning technique, a neural network classifier (more precisely a radial basis function (RBF) network [20]) was trained on the canonical training dataset. The implementation of RBF networks with adaptive centers by [36] was used with default values (*number of centers *3; *regularization *10^-4^;* iterations for optimization *10).

### Assessing performance of classifiers based on three features by jackknife resampling

To estimate the mean accuracy and standard error of the mean of a classifier, it was trained and tested on datasets derived from random splits of the canonical samples derived from Genbank using a 100-fold jackknife resampling process [37]. More specifically, the canonical training and testing datasets described above were concatenated yielding a dataset of 7,604 samples, with 3,802 samples of each class. For each jackknife run, 1,901 samples of each class were chosen randomly from this dataset for training, while the remaining samples were used for testing. The mean accuracy and the standard error of the mean (σ/N
 MathType@MTEF@5@5@+=feaafiart1ev1aaatCvAUfKttLearuWrP9MDH5MBPbIqV92AaeXatLxBI9gBaebbnrfifHhDYfgasaacH8akY=wiFfYdH8Gipec8Eeeu0xXdbba9frFj0=OqFfea0dXdd9vqai=hGuQ8kuc9pgc9s8qqaq=dirpe0xb9q8qiLsFr0=vr0=vr0dc8meaabaqaciaacaGaaeqabaqabeGadaaakeaadaGcaaqaaiabd6eaobWcbeaaaaa@2DEC@, where σ denotes the standard deviation and *N *the number of jackknife resamplings) were calculated.

For each jackknife run, an SVM, RBF network and linear classifier were trained using all three features of the corresponding training dataset. For training the SVM classifier, the kernel parameters as derived by the grid-search on the canonical training dataset (*C *= 12.5; *g *= 6.25) were used. The RBF network was trained using default parameter values (*number of centers *3; *regularization *10^-4^; *iterations for optimization *10). With respect to the linear classifier, threshold calculation by line-sweeping (cf. supplemental Figure S1 [see Additional file 1]) in case of three features cannot be accomplished by an exhaustive search in feasible time, since the search space is cubic. Therefore, we estimated lower and upper bounds and searched for the optimum thresholds within these bounds. To be precise, based on visual inspection (cf. Figure 2) only the following feature ranges were searched by line-sweeping on the training datasets:

1. Sequence similarity: 0.01...0.05

2. Inverse CBIN count: 0.01...0.03

3. Fraction of consecutive matches and mismatches: 0.90...0.94

Although line-sweeping is not exhaustive, the best combination of thresholds found in the reduced search space should represent the optimum; these are 0.01832 for sequence similarity, 0.01613 for inverse CBIN count and 0.92827 for the fraction of consecutive matches and mismatches.

Accuracy, precision and true positive/true negative (TP/TN) and false positive/false negative (FP/FN) ratios were averaged over all jackknife runs and the standard error of the mean of each of these properties was calculated (cf. Table 1 and Figure 4).

### Classifiers based on fewer features, thresholds and parameters; measuring performance

Performance of the classifiers based on three features was compared to the performance of classifiers based on a reduced set of two or only one feature(s), using the canonical training and testing datasets only. In contrast to the studies using resampling, all linear classifiers were derived by exhaustive line sweeping, that is, by an exhaustive search for the best combination of thresholds or the best single threshold in case of one feature. The thresholds for linear classifiers are listed in the supplementary data, Tables S1 and S2 [see Additional file 1]. The kernel parameters (cf. **Methods**, section *Training of the SVM*) for SVM classifiers based on canonical training datasets are listed in Table S3 of the supplementary data [see Additional file 1]. Performance (in terms of accuracy) of all classifiers was noted on canonical testing datasets and homologous-regions-only datasets and is given in Table 3.

## Authors' contributions

MS drafted the manuscript. AS, PC and SL participated in data analysis and helped in drafting the manuscript, together with GF who supervised the research. AS provided all data related to the *Xenopus *ESTs. MS implemented and tested the IsoSVM tool and carried out all classifier training and testing procedures. GF and AS helped with testing and application of the tool.

## Supplementary Material

Additional File 1 Supplementary Material. Threshold levels and kernel parameters used; species affiliation of BLAST query sequences; illustration of the line-sweeping procedure. Click here for file

## Figures and Tables

**Table 1:** Mean accuracy and standard error of the mean of various classifiers, using three features derived from the alignment of the sequences to be compared. 100-fold jackknife resampling was employed. "± " denotes the standard error of the mean.

**SVM classifier**					
**Accuracy**	**Precision**	**True Positives**	**True Negatives**	**False Positives**	**False Negatives**

99.55% ± 0.008	99.31% ± 0.015	1897.1 ± 0.21	1887.9 ± 0.28	13.1 ± 0.28	3.9 ± 0.21

**RBF network classifier**					

**Accuracy**	**Precision**	**True Positives**	**True Negatives**	**False Positives**	**False Negatives**

99.33% ± 0.011	98.91% ± 0.019	1896.5 ± 0.22	1880.1 ± 0.38	20.9 ± 0.38	4.6 ± 0.22

**3-feature linear classifier**					

**Accuracy**	**Precision**	**True Positives**	**True Negatives**	**False Positives**	**False Negatives**

99.42% ± 0.011	99.22% ± 0.020	1893.8 ± 0.35	1886.0 ± 0.39	15.0 ± 0.39	7.2 ± 0.35

**Table 2:** Performance of the SVM classifier (accuracy/precision) on four testing scenarios.

**Full-length-sequence (canonical testing dataset)**					
Accuracy	Precision	True Positives	True Negatives	False Positives	False Negatives

99.63%	99.37%	1899	1889	12	2

**Selected *Xenopus *EST data**					

Accuracy	Precision	True Positives	True Negatives	False Positives	False Negatives

97.93%	99.23%	129	155	1	8
**Homologous-regions-only**					

Accuracy	Precision	True Positives	True Negatives	False Positives	False Negatives

98.98%	97.57%	2529	5455	63	19

**ABC protein homologous-regions-only**					

Accuracy	Precision	True Positives	True Negatives	False Positives	False Negatives

-	95.65%	110	-	5	-

**Table 3:** Performance comparison of the three-feature SVM classifier to linear classifiers, an RBF network classifier and other SVM classifiers, using canonical training and testing datasets.

		**Accuracy**	
		
	**Feature(s)**	**Canonical testing dataset**	**Homologous-regions-only testing dataset**
	
**3-feature SVM classifier**	**Sequence similarity, inverse CBIN count, match/mismatch fraction **(cf. Table 2)	99.63%	98.98%
**2-feature SVM classifiers**	**Match/mismatch fraction, sequence similarity**	97.50%	96.68%
	
	**Inverse CBIN count, sequence similarity**	99.32%	98.97%
	
	**Match/mismatch fraction, inverse CBIN count**	99.42%	98.91%

**RBF Network classifier**	**Sequence similarity, inverse CBIN count, match/mismatch fraction**	99.32%	98.79%

**3-feature linear classifier**	**Sequence similarity, inverse CBIN count, match/mismatch fraction**	99.42%	98.80%

**2-feature linear classifiers**	**Match/mismatch fraction, sequence similarity**	99.03%	98.75%
	
	**Inverse CBIN count, sequence similarity**	99.32%	98.67%
	
	**Match/mismatch fraction, inverse CBIN count**	99.37%	98.77%

**1-feature linear classifiers**	**Sequence similarity**	82.22%	82.02%
	
	**Match/mismatch fraction**	98.05%	98.62%
	
	**Inverse CBIN count**	99.37%	98.75%

**Table 4:** Summary of Xenopus EST cleanup and clustering.

Total number of ESTs and cDNAs	350,468
Number of good sequences	317,242
Average trimmed EST length (bp)	536
Number of clusters	25,971
Number of singletons	40,877
Number of CAP3 contigs	31,353
Number of CAP3 singletons	4,801
Average CAP3 contig length (bp)	1,045
Max. cluster size (no. of ESTs)	6,332
Average cluster size (no. of ESTs)	10.6
**Cluster sizes:**	**# EST**
4,097 – 8,192	1
2,049 – 4,096	1
1,025 – 2,048	2
513 – 1,024	15
257 – 512	35
129 – 256	116
65 – 128	414
33 – 64	973
17 – 32	1,755
9 – 16	2,974
5 – 8	4,571
3 – 4	6,444
2	8,670
