# Supplementary material for: IsoSVM – Distinguishing isoforms and paralogs on the protein level
Source: BMC Bioinformatics. 2006 Mar 6;7:110. doi: 10.1186/1471-2105-7-110 (PMC1431569; doi:10.1186/1471-2105-7-110)
Supplement: Additional File 1 — Supplementary Material. Threshold levels and kernel parameters used; species affiliation of BLAST query sequences; illustration of the line-sweeping procedure. [file 1471-2105-7-110-S1.doc]

# Supplementary data on

# IsoSVM – Distinguishing Isoforms and Paralogs

# on the Protein Level

Michael Spitzer^1^, Stefan Lorkowski^2,3^, Paul Cullen^2^, Alexander Sczyrba^4^, Georg Fuellen^1,5*^

*^1^ Division of Bioinformatics, Biology Department, Schlossplatz 4, 48149 Münster, Germany*

*^2^ Leibniz Institute of Arteriosclerosis Research, Domagkstr. 3, 48149 Münster, Germany*

*^3^ Institute of Biochemistry, Wilhelm-Klemm-Str. 2, 48149 Münster, Germany*

*^4^ Faculty of Technology, Research Group in Practical Computer Science, University of Bielefeld,
Postfach 10 01 31, 33501 Bielefeld, Germany*

*^5^ Department of Medicine, AG Bioinformatics, Domagkstr. 3, 48149 Münster, Germany*

^*^ Corresponding author

**Table S1. Thresholds for linear classifiers based on a single feature.**

| **Feature** | **Threshold** |
| --- | --- |
| **sequence similarity** | 0.57895 |
| **inverse CBIN count** | 0.02500 |
| **match/mismatch fraction** | 0.95190 |

**Table S2. Thresholds for linear classifiers based on a combination of features.**

|  | **Thresholds** | | |
| --- | --- | --- | --- |
| **Feature combination** | **Sequence similarity** | **Match/mis-match fraction** | **Inverse CBIN count** |
| **sequence similarity  + match/mismatch fraction** | 0.13363 | 0.92825 |  |
| **sequence similarity  + inverse CBIN count** | 0.01832 |  | 0.02381 |
| **match/mismatch fraction**  **+**  **inverse CBIN count** |  | 0.92825 | 0.01613 |
| **3-feature linear classifier** | 0.01832 | 0.92827 | 0.01613 |

**Table S3. Kernel parameters for SVM classifiers based on a combination of features.**

|  | **Kernel parameters** | |
| --- | --- | --- |
| **Feature combination** | ***C*** | ***g*** |
| **match/mismatch fraction + sequence similarity** | 10^4^ | 10^3^ |
| **inverse CBIN count**  **+**  **sequence similarity** | 10^4^ | 10^0^ |
| **match/mismatch fraction**  **+**  **inverse CBIN count** | 10^1^ | 10^3^ |
| **3-feature SVM classifier** | 1.25*10^1^ | 6.25*10^0^ |

**Table S4. Species affiliation of the sequences that were used to drive BLAST searches for homologous sequences that have putative splice variants.**

| **Species** | **No. of query sequences** |
| --- | --- |
| *Homo sapiens* | 139 |
| ***Mus musculus*** | 31 |
| ***Caenorhabditis elegans*** | 17 |
| ***Rattus norvegicus*** | 16 |
| ***Bos taurus*** | 3 |
| ***Macaca mulatta*** | 3 |
| ***Arabidopsis thaliana*** | 2 |
| ***Canis familiaris*** | 2 |
| ***Oryctolagus cuniculus*** | 2 |
| ***Drosophila melanogaster*** | 2 |
| ***Xenopus laevis*** | 2 |
| ***Zea mays*** | 2 |
| ***Aedes aegypti*** | 1 |
| ***Ambystoma mexicanum*** | 1 |
| ***Argopecten irradians*** | 1 |
| ***Branchiostoma floridae*** | 1 |
| ***Bubalus bubalis*** | 1 |
| *Canis familiaris* | 1 |
| ***Choristoneura fumiferana*** | 1 |
| ***Cycas revoluta*** | 1 |
| ***Danio rerio*** | 1 |
| ***Drosophila simulans*** | 1 |
| ***Gallus gallus*** | 1 |
| ***Ipomoea batatas*** | 1 |
| ***Lepidosiren paradoxa*** | 1 |
| ***Lycopersicon esculentum*** | 1 |
| ***Macaca fascicularis*** | 1 |
| ***Meleagris gallopavo*** | 1 |
| ***Microcoelia exilis*** | 1 |
| ***Mus sp.*** | 1 |
| ***Mustela putorius furo*** | 1 |
| ***Notechis ater*** | 1 |
| ***Phanerochaete chrysosporium*** | 1 |
| ***Pimephales promelas*** | 1 |
| ***Pseudonaja textilis*** | 1 |
| ***Schistosoma japonicum*** | 1 |
| ***Solanum tuberosum*** | 1 |
| ***Sorghum bicolor*** | 1 |
| ***Spongilla lacustris*** | 1 |
| ***Tenebrio molitor*** | 1 |
| ***Thunnus obesus*** | 1 |
| Total | **250** |


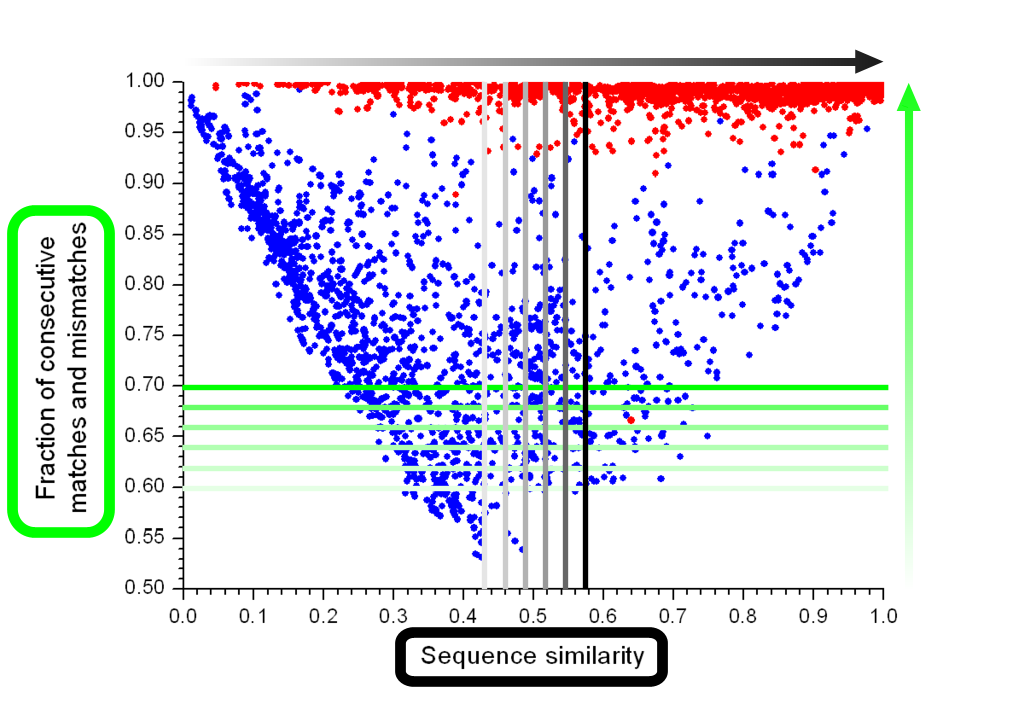


**Figure S1. Illustration of the line-sweeping procedure in the case of classifiers based on two features (here: *sequence similarity* and *fraction of consecutive matches and mismatches*).** A line (= threshold) is swept over the entire range of the first feature, in *n* steps (where *n* is the number of samples in the training dataset; a small offset is added to each value of a feature to prevent the thresholds from being located *on* a sample). For each given threshold for the first feature, the entire range of the second feature is scanned, again in *n* steps. Each pair of thresholds is used to classify the underlying training dataset, and the pair of thresholds with the highest accuracy is denoted the "optimal threshold pair", which is used to classify the corresponding (feature-reduced) testing dataset.
